# Supplementary material for: Relationship between physical activity and ankle osteoarthritis: Implications for metabolic diseases
Source: PLoS One. 2026 May 20;21(5):e0348766. doi: 10.1371/journal.pone.0348766 (PMC13189354; doi:10.1371/journal.pone.0348766)
Supplement: S1 Table — (DOCX) [file pone.0348766.s001.docx]

| Supporting information table 1-1. MET-min statistics stratified by sex and Takakura stage. | | |
| --- | --- | --- |
| Male (n = 98) | Value | Median [IQR] |
| Takakura stage 2 (n = 17) |  |  |
| Total MET-minutes | 2376.9 (SD 2005.5) | 2079 [1074–2754] |
| Vigorous activity MET-minutes | 621.2 (SD 1197.2) | 0 [0–960] |
| Moderate activity MET-minutes | 404.7 (SD 509.5) | 240 [0–480] |
| Walking activity MET-minutes | 1351.1 (SD 1294.4) | 990 [594–1980] |
| Takakura stage 3a (n = 30) |  |  |
| Total MET-minutes | 2761.1 (SD 3314.5) | 1449 [700–3026] |
| Vigorous activity MET-minutes | 704.0 (SD 1625.2) | 0 [0–240] |
| Moderate activity MET-minutes | 497.3 (SD 648.3) | 160 [0–900] |
| Walking activity MET-minutes | 1559.8 (SD 2170.8) | 792 [495–1683] |
| Takakura stage 3b (n = 20) |  |  |
| Total MET-minutes | 2757.0 (SD 3140.5) | 1540 [767–3366] |
| Vigorous activity MET-minutes | 288.0 (SD 769.3) | 0 [0–0] |
| Moderate activity MET-minutes | 654.0 (SD 1235.2) | 0 [0–660] |
| Walking activity MET-minutes | 1815.0 (SD 2732.9) | 974 [619–2153] |
| Takakura stage 4 (n = 31) |  |  |
| Total MET-minutes | 2334.1 (SD 3607.1) | 396 [148–3306] |
| Vigorous activity MET-minutes | 0.0 (SD 0.0) | 0 [0–0] |
| Moderate activity MET-minutes | 1348.4 (SD 2710.8) | 0 [0–1260] |
| Walking activity MET-minutes | 985.7 (SD 1701.5) | 198 [33–842] |

| Supporting information table 1-2. MET-min statistics stratified by sex and Takakura stage. | | |
| --- | --- | --- |
| Female (n = 164) | Value | Median [IQR] |
| Takakura stage 2 (n = 37) |  |  |
| Total MET-minutes | 2862.7 (SD 7110.4) | 924 [198–1413] |
| Vigorous activity MET-minutes | 1528.6 (SD 6010.4) | 0 [0–0] |
| Moderate activity MET-minutes | 177.3 (SD 335.4) | 0 [0–240] |
| Walking activity MET-minutes | 1156.8 (SD 2093.5) | 495 [132–990] |
| Takakura stage 3a (n = 63) |  |  |
| Total MET-minutes | 1677.8 (SD 1689.2) | 924 [462–2494] |
| Vigorous activity MET-minutes | 172.7 (SD 655.5) | 0 [0–0] |
| Moderate activity MET-minutes | 518.7 (SD 1082.8) | 0 [0–720] |
| Walking activity MET-minutes | 986.3 (SD 1037.2) | 693 [396–1287] |
| Takakura stage 3b (n = 32) |  |  |
| Total MET-minutes | 1208.6 (SD 1402.8) | 693 [256–1795] |
| Vigorous activity MET-minutes | 45.0 (SD 187.3) | 0 [0–0] |
| Moderate activity MET-minutes | 491.2 (SD 1123.5) | 0 [0–250] |
| Walking activity MET-minutes | 672.4 (SD 836.3) | 396 [182–693] |
| Takakura stage 4 (n = 32) |  |  |
| Total MET-minutes | 878.5 (SD 1229.1) | 478 [66–1214] |
| Vigorous activity MET-minutes | 0.0 (SD 0.0) | 0 [0–0] |
| Moderate activity MET-minutes | 300.0 (SD 791.9) | 0 [0–0] |
| Walking activity MET-minutes | 578.5 (SD 584.3) | 478 [66–792] |
