# Supplementary material for: Relationship between physical activity and ankle osteoarthritis: Implications for metabolic diseases
Source: PLoS One. 2026 May 20;21(5):e0348766. doi: 10.1371/journal.pone.0348766 (PMC13189354; doi:10.1371/journal.pone.0348766)
Supplement: S2 Table — (DOCX) [file pone.0348766.s002.docx]

| Supporting information table 2. Correlation table between factors in Takakura stage 2 | | | | | | | | | | | |
| --- | --- | --- | --- | --- | --- | --- | --- | --- | --- | --- | --- |
|  | Age | BMI | FAOS_Sx | FAOS_Pain | FAOS_ADL | FAOS_Sports | FAOS_QoL | VAS | Vigorous_MET | Moderate_MET | Walking_MET |
| BMI | -0.064  (p=0.645) |  |  |  |  |  |  |  |  |  |  |
| FAOS_Sx | 0.327^*^  (p=0.016) | 0.095  (p=0.493) |  |  |  |  |  |  |  |  |  |
| FAOS_Pain | 0.090  (p=0.518) | 0.046  (p=0.739) | 0.667^**^  (p<0.001) |  |  |  |  |  |  |  |  |
| FAOS_ADL | -0.158  (p=0.253) | -0.076  (p=0.587) | 0.374^**^  (p=0.005) | 0.795^**^  (p<0.001) |  |  |  |  |  |  |  |
| FAOS_Sports | -0.002  (p=0.987) | 0.004  (p=0.979) | 0.387^**^  (p=0.004) | 0.543^**^  (p<0.001) | 0.671^**^  (p<0.001) |  |  |  |  |  |  |
| FAOS_QoL | -0.023  (p=0.868) | 0.114  (p=0.412) | 0.218  (p=0.113) | 0.466^**^  (p<0.001) | 0.500^**^  (p<0.001) | 0.536^**^  (p<0.001) |  |  |  |  |  |
| VAS | -0.082  (p=0.558) | -0.109  (p=0.432) | -0.384^**^  (p=0.004) | -0.611^**^  (p<0.001) | -0.549^**^  (p<0.001) | -0.484^**^  (p<0.001) | -0.460^**^  (p<0.001) |  |  |  |  |
| Vigorous_MET | -0.202  (p=0.144) | -0.282^*^  (p=0.039) | -0.533^**^  (p<0.001) | -0.267  (p=0.051) | -0.002  (p=0.990) | -0.072  (p=0.603) | -0.108  (p=0.439) | 0.090  (p=0.516) |  |  |  |
| Moderate_MET | 0.010  (p=0.945) | 0.038  (p=0.784) | -0.145  (p=0.295) | -0.061  (p=0.662) | -0.010  (p=0.946) | 0.036  (p=0.798) | -0.124  (p=0.372) | -0.074  (p=0.594) | 0.137  (p=0.324) |  |  |
| Walking_MET | -0.247  (p=0.072) | -0.225  (p=0.102) | -0.319^*^  (p=0.019) | -0.163  (p=0.238) | -0.015  (p=0.916) | 0.004  (p=0.978) | 0.034  (p=0.806) | 0.129  (p=0.353) | 0.324^*^  (p=0.017) | 0.116  (p=0.404) |  |
| Total_MET | -0.246  (p=0.073) | -0.305^*^  (p=0.025) | -0.558^**^  (p<0.001) | -0.280^*^  (p=0.041) | -0.007  (p=0.962) | -0.057  (p=0.681) | -0.088  (p=0.526) | 0.111  (p=0.423) | 0.951^**^  (p<0.001) | 0.220  (p=0.111) | 0.593^**^  (p<0.001) |
| SD = standard deviation; M = male; F = female; FAOS = Foot and Ankle Outcome Score; (Sx = symptom, ADL = activities of daily living, QOL = quality of life); IPAQ = International Physical Activity Questionnaire; MET = Metabolic Equivalent Task minutes  * p < 0.05; ** p = 0.001 | | | | | | | | | | | |
