# Supplementary material for: Relationship between physical activity and ankle osteoarthritis: Implications for metabolic diseases
Source: PLoS One. 2026 May 20;21(5):e0348766. doi: 10.1371/journal.pone.0348766 (PMC13189354; doi:10.1371/journal.pone.0348766)
Supplement: S3 Table — (DOCX) [file pone.0348766.s003.docx]

| Supporting information table 3. Correlation table between factors in Takakura stage 3a | | | | | | | | | | | | |
| --- | --- | --- | --- | --- | --- | --- | --- | --- | --- | --- | --- | --- |
|  | Age | BMI | FAOS_Sx | FAOS_Pain | FAOS_ADL | FAOS_Sports | FAOS_QoL | VAS | Vigorous_MET | Moderate_MET | Walking_MET |  |
| BMI | -0.144  (p=0.170) |  |  |  |  |  |  |  |  |  |  |  |
| FAOS_Sx | 0.152  (p=0.146) | -0.196  (p=0.060) |  |  |  |  |  |  |  |  |  |  |
| FAOS_Pain | 0.066  (p=0.531) | -0.119  (p=0.257) | 0.759^**^  (p<0.001) |  |  |  |  |  |  |  |  |  |
| FAOS_ADL | -0.014  (p=0.895) | -0.123  (p=0.238) | 0.721^**^  (p=0.005) | 0.883^**^  (p<0.001) |  |  |  |  |  |  |  |  |
| FAOS_Sports | 0.008  (p=0.942) | -0.188  (p=0.071) | 0.500^**^  (p<0.001) | 0.665^**^  (p<0.001) | 0.738^**^  (p<0.001) |  |  |  |  |  |  |  |
| FAOS_QoL | 0.029  (p=0.783) | -0.123  (p=0.240) | 0.606^**^  (p<0.001) | 0.688^**^  (p<0.001) | 0.673^**^  (p<0.001) | 0.624^**^  (p<0.001) |  |  |  |  |  |  |
| VAS | 0.021  (p=0.843) | 0.117  (p=0.265) | -0.616^**^  (p<0.001) | -0.717^**^  (p<0.001) | -0.734^**^  (p<0.001) | -0.539^**^  (p<0.001) | -0.633^**^  (p<0.001) |  |  |  |  |  |
| Vigorous_MET | -0.143  (p=0.172) | 0.063  (p=0.550) | 0.242^*^  (p=0.020) | 0.195  (p=0.060) | 0.193  (p=0.063) | 0.097  (p=0.356) | -0.004  (p=0.970) | -0.065  (p=0.535) |  |  |  |  |
| Moderate_MET | 0.134  (p=0.200) | 0.024  (p=0.816) | -0.094  (p=0.368) | -0.047  (p=0.657) | -0.050  (p=0.634) | -0.021  (p=0.844) | 0.012  (p=0.906) | 0.125  (p=0.232) | 0.056  (p=0.597) |  |  |  |
| Walking_MET | -0.042  (p=0.687) | -0.055  (p=0.598) | 0.176  (p=0.091) | 0.112  (p=0.285) | 0.191  (p=0.066) | 0.089  (p=0.396) | 0.060  (p=0.565) | -0.131  (p=0.210) | 0.340^**^  (p=0.001) | 0.007  (p=0.947) |  |  |
| Total_MET | -0.038  (p=0.716) | 0.003  (p=0.974) | 0.185  (p=0.076) | 0.142  (p=0.175) | 0.190  (p=0.068) | 0.093  (p=0.378) | 0.042  (p=0.692) | -0.063  (p=0.550) | 0.697^**^  (p<0.001) | 0.434^**^  (p<0.001) | 0.795^**^  (p<0.001) |  |
| SD = standard deviation; M = male; F = female; FAOS = Foot and Ankle Outcome Score; (Sx = symptom, ADL = activities of daily living, QOL = quality of life); IPAQ = International Physical Activity Questionnaire; MET = Metabolic Equivalent Task minutes  * p < 0.05; ** p = 0.001 | | | | | | | | | | | |  |
