# Supplementary material for: Relationship between physical activity and ankle osteoarthritis: Implications for metabolic diseases
Source: PLoS One. 2026 May 20;21(5):e0348766. doi: 10.1371/journal.pone.0348766 (PMC13189354; doi:10.1371/journal.pone.0348766)
Supplement: S4 Table — (DOCX) [file pone.0348766.s004.docx]

| Supporting information table 4. Supporting information table 4. Correlation table between factors in Takakura stage 3b | | | | | | | | | | | |
| --- | --- | --- | --- | --- | --- | --- | --- | --- | --- | --- | --- |
|  | Age | BMI | FAOS_Sx | FAOS_Pain | FAOS_ADL | FAOS_Sports | FAOS_QoL | VAS | Vigorous_MET | Moderate_MET | Walking_MET |
| BMI | -0.060  (p=0.675) |  |  |  |  |  |  |  |  |  |  |
| FAOS_Sx | 0.268  (p=0.054) | -0.005  (p=0.971) |  |  |  |  |  |  |  |  |  |
| FAOS_Pain | 0.275^*^  (p=0.048) | -0.090  (p=0.527) | 0.757^**^  (p<0.001) |  |  |  |  |  |  |  |  |
| FAOS_ADL | 0.137  (p=0.334) | -0.107  (p=0.449) | 0.701^**^  (p=0.005) | 0.853^**^  (p<0.001) |  |  |  |  |  |  |  |
| FAOS_Sports | -0.095  (p=0.502) | 0.041  (p=0.773) | 0.406^**^  (p=0.003) | 0.533^**^  (p<0.001) | 0.695^**^  (p<0.001) |  |  |  |  |  |  |
| FAOS_QoL | -0.036  (p=0.802) | -0.269  (p=0.054) | 0.354^**^  (p=0.010) | 0.511^**^  (p<0.001) | 0.614^**^  (p<0.001) | 0.592^**^  (p<0.001) |  |  |  |  |  |
| VAS | -0.075  (p=0.596) | 0.148  (p=0.294) | -0.475^**^  (p<0.001) | -0.728^**^  (p<0.001) | -0.768^**^  (p<0.001) | -0.596^**^  (p<0.001) | -0.686^**^  (p<0.001) |  |  |  |  |
| Vigorous_MET | -0.325^*^  (p=0.019) | 0.051  (p=0.719) | 0.136  (p=0.335) | 0.303^*^  (p=0.029) | 0.297^*^  (p=0.045) | 0.252  (p=0.072) | 0.339^*^  (p=0.014) | -0.283^*^  (p=0.042) |  |  |  |
| Moderate_MET | -0.060  (p=0.674) | 0.019  (p=0.892) | 0.200  (p=0.156) | 0.117  (p=0.410) | 0.173  (p=0.221) | 0.266  (p=0.056) | 0.280^*^  (p=0.044) | -0.240  (p=0.086) | -0.017  (p=0.908) |  |  |
| Walking_MET | 0.076  (p=0.592) | -0.216  (p=0.124) | 0.172  (p=0.222) | 0.238  (p=0.090) | 0.246  (p=0.079) | 0.202  (p=0.150) | 0.138  (p=0.330) | -0.105  (p=0.459) | 0.166  (p=0.239) | 0.007  (p=0.959) |  |
| Total_MET | -0.039  (p=0.785) | -0.153  (p=0.279) | 0.267  (p=0.055) | 0.315^*^  (p=0.023) | 0.344^*^  (p=0.013) | 0.349^*^  (p=0.011) | 0.323^*^  (p=0.019) | -0.265  (p=0.058) | 0.342^*^  (p=0.013) | 0.499^**^  (p<0.001) | 0.844^**^  (p<0.001) |
| SD = standard deviation; M = male; F = female; FAOS = Foot and Ankle Outcome Score; (Sx = symptom, ADL = activities of daily living, QOL = quality of life); IPAQ = International Physical Activity Questionnaire; MET = Metabolic Equivalent Task minutes  * p < 0.05; ** p = 0.001 | | | | | | | | | | | |
