# Supplementary material for: Relationship between physical activity and ankle osteoarthritis: Implications for metabolic diseases
Source: PLoS One. 2026 May 20;21(5):e0348766. doi: 10.1371/journal.pone.0348766 (PMC13189354; doi:10.1371/journal.pone.0348766)
Supplement: S5 Table — (DOCX) [file pone.0348766.s005.docx]

| Supporting information table 5. Correlation table between factors in Takakura stage 4 | | | | | | | | | | | |
| --- | --- | --- | --- | --- | --- | --- | --- | --- | --- | --- | --- |
|  | Age | BMI | FAOS_Sx | FAOS_Pain | FAOS_ADL | FAOS_Sports | FAOS_QoL | VAS | Vigorous_MET | Moderate_MET | Walking_MET |
| BMI | 0.026  (p=0.840) |  |  |  |  |  |  |  |  |  |  |
| FAOS_Sx | 0.289^*^  (p=0.022) | 0.125  (p=0.330) |  |  |  |  |  |  |  |  |  |
| FAOS_Pain | -0.113  (p=0.380) | 0.089  (p=0.489) | 0.646^**^  (p<0.001) |  |  |  |  |  |  |  |  |
| FAOS_ADL | -0.377^**^  (p=0.002) | 0.087  (p=0.498) | 0.422^**^  (p=0.001) | 0.785^**^  (p<0.001) |  |  |  |  |  |  |  |
| FAOS_Sports | -0.260^*^  (p=0.039) | -0.040  (p=0.753) | 0.310^*^  (p=0.013) | 0.487^**^  (p<0.001) | 0.617^**^  (p<0.001) |  |  |  |  |  |  |
| FAOS_QoL | -0.194  (p=0.128) | 0.034  (p=0.794) | 0.404^**^  (p=0.001) | 0.585^**^  (p<0.001) | 0.581^**^  (p<0.001) | 0.535^**^  (p<0.001) |  |  |  |  |  |
| VAS | 0.249^*^  (p=0.049) | -0.153  (p=0.232) | -0.384^**^  (p=0.002) | -0.720^**^  (p<0.001) | -0.650^**^  (p<0.001) | -0.464^**^  (p<0.001) | -0.673^**^  (p<0.001) |  |  |  |  |
| Vigorous_MET | NA | NA | NA | NA | NA | NA | NA | NA |  |  |  |
| Moderate_MET | 0.010  (p=0.940) | -0.166  (p=0.193) | -0.113  (p=0.380) | -0.072  (p=0.574) | -0.008  (p=0.952) | 0.077  (p=0.547) | 0.120  (p=0.351) | 0.079  (p=0.538) | NA |  |  |
| Walking_MET | 0.121  (p=0.343) | -0.098  (p=0.444) | 0.100  (p=0.433) | 0.108  (p=0.399) | 0.133  (p=0.299) | 0.340^**^  (p=0.006) | 0.265^*^  (p=0.036) | -0.139  (p=0.277) | NA | 0.353^**^  (p=0.005) |  |
| Total_MET | 0.063  (p=0.623) | -0.168  (p=0.188) | -0.037  (p=0.774) | -0.004  (p=0.978) | 0.056  (p=0.665) | 0.214  (p=0.092) | 0.210  (p=0.098) | -0.006  (p=0.965) | NA | 0.902^**^  (p<0.001) | 0.722^**^  (p<0.001) |
| SD = standard deviation; M = male; F = female; FAOS = Foot and Ankle Outcome Score; (Sx = symptom, ADL = activities of daily living, QOL = quality of life); IPAQ = International Physical Activity Questionnaire; MET = Metabolic Equivalent Task minutes  * p < 0.05; ** p = 0.001 | | | | | | | | | | | |
