# Supplementary material for: Relationship between physical activity and ankle osteoarthritis: Implications for metabolic diseases
Source: PLoS One. 2026 May 20;21(5):e0348766. doi: 10.1371/journal.pone.0348766 (PMC13189354; doi:10.1371/journal.pone.0348766)
Supplement: Attachment — Submitted filename: 3. point by point response reviewer.docx [file pone.0348766.s007.docx]

**Point-by-point Response**

**Response to Editor and Reviewers**

**Manuscript title: Relationship Between Physical Activity and Ankle Osteoarthritis: Implications for metabolic diseases**

**Journal: PLOS ONE**

**Manuscript ID: PONE-D-25-50381**

**Dear Academic Editor and Reviewers,**

We fully agree with your comments. We revised the manuscript to address all points, ensured that results are accurately reported, standardized IPAQ-SF terminology, strengthened statistical reporting for skewed/zero-inflated MET-min/week, clarified multiple testing, expanded limitations, toned down potentially overstated interpretations, and audited references to ensure that each citation directly supports the corresponding statement.

Below, we provide a point-by-point response.

**1) Editor Comments**

**Comment (Academic Editor)**

“Please make necessary corrections based on the comments provided by the reviewers.”

**Response**

We fully agree with your comment. We carefully revised the manuscript according to all reviewer reports. Major revisions include: (1) standardized IPAQ-SF terminology (walking/moderate/vigorous) and correction of table/figure typos; (2) added subgroup descriptive statistics for MET-min/week stratified by sex and Takakura stage (Supporting Information 1); (3) clarified structural-zero issues for vigorous activity in Takakura stage 4; (4) expanded limitations and revised conclusions to avoid overgeneralization; and (5) corrected mismatched citations.

**Changes made**

“We standardized IPAQ-SF terminology throughout the manuscript to walking, moderate, vigorous, and total MET-min/week.”

“We added sex- and stage-stratified descriptive statistics for MET-min/week in Supporting Information (S1 Table) to improve interpretability of the correlation analyses.”

“We revised the Discussion and Conclusion to avoid causal language and to reflect the modest magnitude of observed associations.”

“We audited the reference list and corrected citations that did not directly support the corresponding statements.”

**2) Journal Requirements (PLOS ONE)**

**Requirement 1**

1. Please ensure that your manuscript meets PLOS ONE's style requirements, including those for file naming. The PLOS ONE style templates can be found at

https://journals.plos.org/plosone/s/file?id=wjVg/PLOSOne_formatting_sample_main_body.pdf and

**Response**

We fully agree. We revised the manuscript to meet PLOS ONE style requirements and enabled continuous line numbering to facilitate review.

**Requirement 2**

Please include your tables as part of your main manuscript and remove the individual files. Please note that supporting information tables (should remain/ be uploaded) as separate "supporting information" files.

**Response**

We fully agree. We inserted all main tables into the manuscript file in read order after first citation. To improve readability, we moved large stage-stratified correlation matrices to Supporting Information and labeled them as exploratory.

**Requirement 3**

We note that your Data Availability Statement is currently as follows: If the data are all contained within the manuscript and/or Supporting Information files, enter the following: All relevant data are within the manuscript and its Supporting Information files.

**Response**

We fully agree. We confirm that we now provide the minimal de-identified dataset required to replicate all findings reported in the manuscript, along with a codebook/metadata file.

**Requirement 4**

Please amend either the title on the online submission form (via Edit Submission) or the title in the manuscript so that they are identical.

**Response**

We fully agree. We ensured that the title in the submission system and the title on the manuscript title page are identical.

**Requirement 5**

If the reviewer comments include a recommendation to cite specific previously published works, please review and evaluate these publications to determine whether they are relevant and should be cited. There is no requirement to cite these works unless the editor has indicated otherwise.

**Response**

We fully agree. We audited the references and corrected mismatched citations to ensure that each reference supports the corresponding statement.

**3) Reviewer #1**

**Comment 1**

Limitations to be acknowledged in the discussion or remedied: As a cross section study the observed associations do not establish directionality. There is a risk of selection bias as the study was conducted at a tertiary referral center, the cohort may represent more severe cases, limiting generalizability. The self-reported physical activity is subject to recall and social desirability bias. There is no comparison with healthy controls or patients with other types of arthritis limits context for the findings.
**Response**

We fully agree with your comment. We expanded the Discussion to explicitly acknowledge the cross-sectional design, potential selection bias from the tertiary referral setting, limitations of self-reported IPAQ-SF, and the lack of healthy controls or other arthritis comparator groups.

**Changes made**

Line [286–308]

This study has several limitations. First, the retrospective cross-sectional design does not allow inference of causality or directionality between ankle osteoarthritis severity and physical activity. Second, the tertiary referral setting and the absence of a healthy control or other-arthritis comparator may introduce selection bias and limit generalizability and contextual interpretation. In addition, cardiometabolic comorbidities and metabolic outcomes were not systematically assessed; thus, any cardiometabolic implications should be considered hypothesis-generating. Third, physical activity was assessed using the self-reported IPAQ-SF and may be affected by recall and social desirability bias; moreover, MET-min/week variables were right-skewed with many zero values (especially vigorous activity). In Takakura stage 4, vigorous activity was uniformly absent, creating a floor effect; therefore, total MET-min/week (walking + moderate activity) was used to represent overall activity volume in this subgroup, although intensity-specific associations could not be evaluated. Accordingly, correlation-based estimates may have been influenced by non-normality, zero-inflation, and outliers; Fourth, while Holm–Bonferroni adjustment was applied within each correlation table, multiple related subgroup and exploratory analyses were conducted across several tables; thus, stage-stratified findings should be interpreted as exploratory given the possibility of residual multiplicity. Finally, the effect of medical treatment was not considered and may have influenced FAOS scores. Future prospective or case-control studies using objective activity measures (e.g., accelerometers) and cardiometabolic endpoints are warranted to reduce self-report bias and better evaluate the directionality of observed associations.

**Comment 2**

Some of the terminologies are inconsistent: some tables use “light activity” while IPAQ-SF typically uses “walking”; clarity is needed.

**Response**

We fully agree with your comment. We standardized terminology to match IPAQ-SF (walking, moderate, vigorous) throughout the manuscript and tables.

**Changes made**

We have revised “Light activity MET” to “Walking MET” throughout the manuscript and in all tables.

**Comment 3**

Was the MET data normally distributed? The MET-minute data (especially vigorous activity) mean total MET = 2021.6, SD = 3476.6 suggests right skew. Were transformations or robust methods considered?

**Response**

We fully agree with your comment.

**Changes made**

“Because IPAQ-derived MET-min/week variables were right-skewed with many zero values (particularly vigorous activity), we additionally report median (IQR) for MET-min/week outcomes (Table 1.) and interpret correlation-based findings cautiously.

We note in the limitations that non-normality/zero-inflation and outliers may influence correlation-based inference and that future studies should consider rank-based or other robust methods.”

**Comment 4**

“Clarify how multiple testing was handled across Tables 2–8.”

**Response**

We fully agree with your comment. A similar concern was also raised by another reviewer regarding the interpretability and rigor of the analyses in the presence of skewed IPAQ-derived MET-min/week data and multiple subgroup results.

**Changes made**

Line [298-301] Fourth, while Holm–Bonferroni adjustment was applied within each correlation table, multiple related subgroup and exploratory analyses were conducted across several tables; thus, stage-stratified findings should be interpreted as exploratory given the possibility of residual multiplicity.

**Comment 5**

“Provide MET summary statistics by Takakura stage and gender.”

**Response**

We fully agree with your comment. We added a supporting table reporting MET-min/week stratified by sex and Takakura stage.

**Changes made**

“S1 Table. MET-min/week summary statistics (mean ± SD and median [IQR]) stratified by sex and Takakura stage.”

**Comment 6**

In Takakura stage 4, no patients reported vigorous activity, making correlations impossible. This is noted, but the analysis could be refined—e.g., combining moderate + walking METs for that group.

**Response**

We fully agree with your comment. Because no vigorous activity was reported, total MET (calculated as the sum of walking activity MET and moderate activity MET) was included in the analysis. We will add the following statement to the Limitations section.

**Changes made**

Line [294-297] In Takakura stage 4, vigorous activity was uniformly absent, creating a floor effect; therefore, total MET-min/week (walking + moderate activity) was used to represent overall activity volume in this subgroup, although intensity-specific associations could not be evaluated.

**Comment 7**

“No Adjustment for Multiple Testing Across All Tables. While Holm–Bonferroni was applied within tables, there are 8 tables total, increasing the family-wise error rate across the study. Suggestion: Acknowledge this in limitations or apply a stricter correction across all primary outcomes.”
**Response**

We fully agree with your comment. We clarified prespecified outcomes vs exploratory analyses and added an explicit limitation that exploratory subgroup analyses may be susceptible to residual multiplicity.

**Changes made**

Line [298-301] Fourth, while Holm–Bonferroni adjustment was applied within each correlation table, multiple related subgroup and exploratory analyses were conducted across several tables; thus, stage-stratified findings should be interpreted as exploratory given the possibility of residual multiplicity.”

**Comment 8**

“Pain VAS was not correlated with MET-minutes in many groups, yet it was included in all models. The rationale should be stated.”
**Response**

We fully agree with your comment. We clarified the rationale for including pain VAS a priori. The relationship between pain and physical activity is complex and potentially bidirectional: some patients may reduce activity because of pain, whereas others may experience increased pain as a consequence of activity. In addition, analgesic or anti-inflammatory medications and other treatments may reduce pain and thereby modify activity levels, and these factors may not be fully captured in a retrospective design. Despite the absence of statistically significant correlations between pain VAS and MET-minutes in some subgroups, pain remains a clinically important determinant of activity limitation and is closely linked to osteoarthritis severity; therefore, we included pain VAS as a prespecified covariate to account for differences in pain burden when evaluating the association between radiographic severity and physical activity. We also expanded the limitations to acknowledge that, due to the retrospective cross-sectional design and incomplete treatment-related information, residual confounding and the temporal sequence among pain, treatment, and activity cannot be determined and future prospective longitudinal studies are warranted.

**Changes made**

Line [167-169] “Pain severity (VAS) was included a priori as a covariate because pain is a key clinical determinant of functional limitation and may confound the association between osteoarthritis severity and physical activity. “

Line [303-305] Because pain, treatment exposure, and physical activity may interact in a bidirectional and time-dependent manner, the retrospective cross-sectional design cannot determine temporal sequence and residual confounding may remain

**Comment 9**

“No mention of checking for multicollinearity (e.g., VIF) or linearity assumptions in partial correlations.”

**Response**

We fully agree with your comment. We assessed multicollinearity among covariates (age, sex, BMI, and VAS) by calculating variance inflation factors (VIF) in multivariable regression models for each FAOS subscale. Using VIF < 5 as the criterion, no concerning collinearity was identified (all VIF ≤ 1.017). We added this information to the Methods and summarized the results accordingly.

**Changes made**

Line [162-164] “Multivariable linear regression models were fitted for each FAOS subscale (Symptoms, Pain, Activities of Daily Living, Sports/Recreation, and Quality of Life) including age, sex, BMI, and pain severity (VAS) as covariates. Multicollinearity among covariates was assessed using variance inflation factors (VIF), with VIF < 5 considered acceptable, and no concerning collinearity was observed (all VIF ≤ 1.017).”

Line [209-212] “In multivariable models adjusting for age, sex, and BMI, higher pain severity (VAS) was consistently associated with worse scores across all FAOS subscales (all p < 0.001). Subsequently, stage-stratified analyses showed the following associations between FAOS subscales and MET-min/week (supporting inform Tables 2–5).”

**Suggestion 1**

“Introduction & Discussion: State explicitly the hypothesized mechanistic pathway between ankle OA → reduced PA → metabolic disease risk. Discuss how findings align or contrast with existing literature on knee/hip OA and metabolic health.”

**Response**

We fully agree with your suggestion. We revised the Introduction and Discussion to explicitly describe the hypothesized pathway whereby greater ankle osteoarthritis severity may be associated with reduced physical activity, which may in turn be relevant to cardiometabolic risk. We also added a brief discussion comparing our findings with prior literature on knee and hip osteoarthritis, physical activity, and metabolic health, highlighting similarities and differences in observed associations.

**Changes made**

Line [83-85] “We hypothesized that greater ankle osteoarthritis severity would be associated with lower habitual physical activity, and that reduced activity may be relevant to cardiometabolic health.”

Line [232–234] “These results are broadly consistent with prior studies in knee and hip osteoarthritis reporting lower physical activity with greater disease burden, although patterns may differ by joint and patient characteristics.”

**Suggestion 2**

“Improve Data Presentation: Correct table typos (“Thakstan” vs. “Takakura”) and ensure consistent terminology. Consider consolidating some correlation tables or moving them to supplementary materials to improve readability. For example: Tables 2–4 (Partial correlations by whole group, male, female) could be consolidated into one table with clear column labeling for subgroups. Tables 5–8 (Correlations by Takakura stage) could either be combined into one summary table highlighting only significant correlations, or moved to Supplementary Materials.”

**Response**

We fully agree with your suggestion. We corrected typographical errors (e.g., “Thakstan” to “Takakura”) and standardized activity terminology to align with the IPAQ-SF (walking, moderate, vigorous) across the manuscript and tables. To improve readability, we streamlined the presentation of correlation results by moving the stage-stratified correlation tables (Tables 5–8) to the Supporting Information and summarizing the key findings in the main Results.

Changes made

“Typographical errors were corrected (e.g., ‘Thakstan’ revised to ‘Takakura’) and activity labels were standardized to ‘walking,’ ‘moderate,’ and ‘vigorous.”

Line [213-214] “Detailed stage-stratified correlation results are provided in S2–S5 Tables (Supporting Information), and key findings are summarized.”

Supporting Information captions

“S2 Table. Correlation table between factors in Takakura stage 2”

“S3 Table. Correlation table between factors in Takakura stage 3a”

“S4 Table. Correlation table between factors in Takakura stage 3b.”

“S5 Table. Correlation table between factors in Takakura stage 4.”

**Suggestion 3**

“Expand in discussion of limitation on how future studies could overcome the limitations of self-reporting (e.g., using accelerometers). A prospective or case-control design could better establish causality.”

**Response**

We fully agree with your suggestion. We expanded the Limitations section to note that self-reported physical activity may be subject to measurement bias and that future studies should incorporate objective activity measures (e.g., accelerometers) and prospective or case-control designs to better address directionality and causality.

**Changes made**

Line [305–308] “Future prospective or case-control studies using objective activity measures (e.g., accelerometers) and cardiometabolic endpoints are warranted to reduce self-report bias and better evaluate the directionality of observed associations.”

**Suggestion 4**

“Refine the Conclusion: Make the ‘take-home’ message clearer regarding clinical implications. Should ankle OA management include PA promotion to mitigate metabolic risk?”

**Response**

We fully agree with your suggestion. We revised the Conclusion to provide a clearer take-home message while maintaining an appropriately cautious interpretation. We emphasize that an association between ankle osteoarthritis severity and physical activity was observed, and that these findings may support the relevance of activity-focused assessment and counseling, while noting that causality cannot be inferred from the current design.

**Changes made**

Line [311–318] (Conclusion)

In this single-center, retrospective cross-sectional study, physical activity levels measured by MET-minutes were associated with radiographic ankle osteoarthritis severity and demographic factors such as age and BMI, with greater radiographic severity corresponding to lower self-reported physical activity. These associations were modest and should be interpreted as observational rather than causal; accordingly, the current design cannot determine directionality or clarify potential metabolic implications. Future prospective studies incorporating objective activity monitoring and cardiometabolic endpoints are warranted to assess whether ankle osteoarthritis contributes to metabolic risk and to evaluate whether treatment influences physical activity and systemic health outcomes.

**Suggestion 5**

“Consider a Visual Abstract or Summary Figure: A conceptual diagram linking ankle OA, PA, and metabolic disease could enhance reader engagement and clarity.”

**Response**

We agree that a conceptual summary may enhance clarity. We added a schematic figure illustrating the hypothesized relationships among ankle osteoarthritis severity, physical activity, and cardiometabolic health for reader accessibility.

**Changes made**

The hypothesized pathway linking ankle osteoarthritis severity, reduced physical activity, and potential cardiometabolic implications (Figure 2).

Figure of legend 2 Figure 2. Conceptual diagram illustrating the hypothesized pathway linking radiographic ankle osteoarthritis severity to reduced physical activity and potential cardiometabolic implications. Greater ankle osteoarthritis severity may be associated with increased pain and stiffness and reduced mobility, which may contribute to lower habitual physical activity (MET-min/week). Reduced physical activity may in turn be related to decreased energy expenditure and fitness, with potential downstream associations with adverse cardiometabolic health outcomes (e.g., obesity, insulin resistance, and cardiovascular risk).

**Suggestion 6**

“Some references (e.g., #6 on cancer) seem misplaced; ensure all citations directly support the manuscript’s claims.”

**Response**

We fully agree with your suggestion. We reviewed all references for relevance and corrected or removed citations that did not directly support the associated statements, ensuring that each citation appropriately matches the claim in the text.

**Changes made**

“We reviewed and revised the reference list to ensure that all citations directly support the corresponding statements in the manuscript.”

**4) Reviewer #2**

**Comment 1 (Introduction)**

“Expand the statements on ankle osteoarthritis and what is the cause of this pathology.”

**Response**

We fully agree with your comment. We expanded the Introduction to describe ankle OA etiology and key mechanical contributors.

**Changes made**

Line [58–61] “Unlike hip or knee osteoarthritis, is most commonly post-traumatic, developing after prior ankle fractures or ligament injuries. Mechanical factors such as joint incongruity, instability, and malalignment contribute to disease onset and progression and may substantially restrict mobility and physical activity.”

**Comment 2 (Introduction)**

“Expand the study objective. The pathology and physical activity can be more detailed. As written it appears yes/no for pathology.
**Response**

We fully agree with your comment. We clarified that OA severity and physical activity were assessed on continuous/ordinal scales rather than binary measures.

**Changes made**

Line [80–83] The objective of this study was to examine the association between radiographic ankle osteoarthritis severity (Takakura stage) and habitual physical activity quantified using METs (Metabolic Equivalent of Task), and to explore how these relationships vary by demographic and clinical characteristics.

**Comment 3 (Methods)**

“The authors discuss the disease state that can result from lack of activity brought on by ankle OA. Other medical history data would support the objective of the study to determine the systemic level of disease.”

**Response**

We fully agree with your comment. Our study aimed to examine whether ankle osteoarthritis severity and related symptoms are associated with patients’ physical activity levels and to discuss the potential cardiometabolic relevance of reduced activity; however, this was not a study specifically enrolling or analyzing patients with confirmed metabolic diseases. In this retrospective dataset, we did not comprehensively assess underlying cardiometabolic comorbidities or relevant medical history (e.g., diabetes, hypertension, dyslipidemia). Therefore, we revised the manuscript to avoid implying direct evaluation of systemic metabolic disease and explicitly acknowledged the lack of detailed comorbidity data as a key limitation. Accordingly, any metabolic implications are framed as hypothesis-generating and should be confirmed in future longitudinal studies incorporating cardiometabolic endpoints.

**Changes made**

**Line [290–292]** In addition, cardiometabolic comorbidities and metabolic outcomes were not systematically assessed; thus, any cardiometabolic implications should be considered hypothesis-generating.

**Comment 4 (Methods)**

“While trying to maintain a homogenous sample, the exclusion criteria appear to have potential impact on the results. Why eliminate variables that would impact physical activity and other severities? there are also subsets of patients that could be analyzed and provide a larger impact on the results.”

**Response**

We fully agree with your comment. We clarified the rationale for our inclusion and exclusion criteria and acknowledged their potential impact on generalizability. Because this study focused on radiographic ankle osteoarthritis, we included only patients with definite radiographic OA, defined as modified Takakura stage ≥2. We excluded Takakura stage 1 because stage 1 typically does not show definite radiographic osteoarthritic changes and may represent early or ambiguous findings; therefore, restricting inclusion to stage ≥2 improved internal validity and ensured a more homogeneous radiographic OA cohort for evaluating the association between radiographic severity and physical activity. We also clarified that additional exclusion criteria (e.g., neuromuscular disease, inflammatory arthritis, avascular necrosis, non-ambulatory status, and valgus-type OA) were applied to reduce confounding from conditions with distinct pathophysiology or independent mobility limitations.

**Comment 5 (Methods)**

“The statistical analysis is very basic. There are other questions that can asked of the data to beyond correlations. Consider stratification based on the clinical measures.”

**Response**

We fully agree with your comment. A similar concern was also raised by another reviewer regarding the interpretability and rigor of the analyses in the presence of skewed IPAQ-derived MET-min/week data and multiple subgroup results. Accordingly, we strengthened the statistical reporting and methodological checks in three ways. First, because MET-min/week variables (particularly vigorous activity) are right-skewed with many zero values, we additionally report median and interquartile range (IQR; 25th–75th percentiles) to complement mean (SD) and improve interpretability. Second, to address the request for stratification, we provided sex- and Takakura stage–stratified descriptive statistics for MET-min/week in Supporting Information and explicitly stated that stage-stratified analyses are exploratory. Third, we assessed multicollinearity among covariates (age, sex, BMI, and VAS) by calculating variance inflation factors (VIF) in multivariable regression models for each FAOS subscale. Using VIF < 5 as the criterion, no concerning collinearity was identified (all VIF ≤ 1.017). We added these clarifications and checks to the Methods and updated the Results/Supporting Information accordingly.

**Changes made**

Line [162-164] “Multivariable linear regression models were fitted for each FAOS subscale (Symptoms, Pain, Activities of Daily Living, Sports/Recreation, and Quality of Life) including age, sex, BMI, and pain severity (VAS) as covariates. Multicollinearity among covariates was assessed using variance inflation factors (VIF), with VIF < 5 considered acceptable, and no concerning collinearity was observed (all VIF ≤ 1.017).”

Line [209-212] “In multivariable models adjusting for age, sex, and BMI, higher pain severity (VAS) was consistently associated with worse scores across all FAOS subscales (all p < 0.001). Subsequently, stage-stratified analyses showed the following associations between FAOS subscales and MET-min/week (Supporting Tables S 2–5).”

Line [448–449] Supporting information table1.

Table 1-1 (male), Table 1-2 (female) MET-min/week summary statistics (mean ± SD and median [IQR]) stratified by sex and Takakura stage.

**Comment 6–7 (Results)**

Line 164 Provide data mean (SD) in table to support the correlations.

**Response**

We fully agree with your comment. We added descriptive statistics (mean ± SD and median [IQR]) for MET-min/week to provide the values underlying correlation analyses.

**Changes made**

Line [179–181] “Baseline characteristics are shown in Table 1, and sex- and Takakura stage–stratified MET-min/week summary statistics (mean ± SD and median [IQR]) are provided in S1 Table.”

**Comment**

Line 166 Data are provided for total enrollment of males vs females but no other stratification; results should be stated for overall, males, and females.

**Response**

We fully agree with your comment. We provided sex-stratified summaries by Takakura stage in S1 Table and clearly separated overall and sex-stratified analyses in the Results.

**Changes made**

Line [179–181] “Baseline characteristics are shown in Table 1, and sex- and Takakura stage–stratified MET-min/week summary statistics (mean ± SD and median [IQR]) are provided in Supporting information table 1 (S1 table).”

Line [192–193] “To improve clarity, we present overall adjusted associations in Table 2 and sex-stratified adjusted associations in Tables 3 and 4.”

**Comment**:

Line 170 What analyses were used for comparisons between males and females for MET-minutes? Why not include group comparisons for sex?

**Response**

We fully agree with your comment. We explicitly stated the statistical tests used for male–female comparisons.

**Changes made**

Line [185–186] “Comparisons between males and females were performed using the independent t-test for continuous variables, and the chi-square test for categorical variables.”

**Comment**:

Line 175 Redundancy/unclear distinction between overall vs sex-specific findings.

**Response**

We fully agree with your comment. We reorganized the Results to clearly distinguish overall findings, sex comparisons, and sex-stratified adjusted analyses, and removed redundant wording. At the same time, because the relative clinical relevance of different activity-intensity metrics (e.g., vigorous versus total MET-min/week) to potential cardiometabolic implications in ankle osteoarthritis remains uncertain, we reported associations for the full set of MET outcomes (walking, moderate, vigorous, and total) rather than selectively presenting only one metric. Notably, the sex-stratified analyses showed that total MET-min/week was associated with radiographic severity in females, whereas vigorous MET-min/week showed an association in males; therefore, presenting both vigorous and total MET-min/week helps avoid premature prioritization of a single activity metric.

**Changes made**

Line [192–193] “To improve clarity, we present overall adjusted associations in Table 2 and sex-stratified adjusted associations in Tables 3 and 4.”

**Comment 8–9 (Discussion/Conclusion)**

**Comment**:

Line 206 Findings are overgeneralized; correlations are weak.

**Response**

We fully agree with your comment. We revised the Discussion to emphasize that the observed associations are modest in magnitude and should be interpreted as observational.

**Changes made**

Line [231–232] These associations were of limited strength and should be interpreted as observational rather than causal.

**Comment:**

Line 211 Why only discuss the female cohort?

**Response**

We agree with your comment. We performed sex-stratified analyses for both males and females and reported results for both subgroups. In the Discussion, we placed relatively greater emphasis on the female cohort because the observed associations were more consistent and statistically apparent in females, whereas associations were weaker and less consistent in males. However, we acknowledge that subgroup findings may be influenced by sample size and limited power and should be interpreted as exploratory; we revised the Discussion to reflect this and to provide a more balanced interpretation across sexes.

**Changes made**

Line [253–256] Although we present sex-stratified findings for both males and females, associations were more consistent in females in our dataset; therefore, interpretations remain cautious and subgroup findings should be considered exploratory given potential differences in sample size and statistical power.

**Comment:**

Line 218 Systemic diseases referenced but not presented; recommend extracting and including in analyses.

**Response**

We fully agree with your comment. Cardiometabolic comorbidities and metabolic outcomes were not systematically available in this retrospective dataset and were therefore not included in the analyses; we clarified this and added it as a key limitation.

**Changes made**

Line [290–292] “In addition, cardiometabolic comorbidities and metabolic outcomes were not systematically assessed; thus, any cardiometabolic implications should be considered hypothesis-generating.”

**Comment:**

Conclusion overstated; focus only on correlations; weak relationships; other data needed.

**Response**

We fully agree with your comment. We revised the Conclusion to avoid overstatement by limiting the interpretation to associations/correlations only, explicitly noting that the observed relationships were modest in strength and observational, and that causality and directionality cannot be inferred from the current cross-sectional design. We also added that additional prospective studies with objective activity monitoring and relevant metabolic endpoints are needed to confirm and extend these findings.

**Changes made**

Line [311–318] (Conclusion)

“In this single-center, retrospective cross-sectional study, physical activity levels measured by MET-minutes were associated with radiographic ankle osteoarthritis severity and demographic factors such as age and BMI, with greater radiographic severity corresponding to lower self-reported physical activity. These associations were modest and should be interpreted as observational rather than causal; accordingly, the current design cannot determine directionality or clarify potential metabolic implications. Future prospective studies incorporating objective activity monitoring and cardiometabolic endpoints are warranted to assess whether ankle osteoarthritis contributes to metabolic risk and to evaluate whether treatment influences physical activity and systemic health outcomes.
